# Bridging Gaps in *Panthera leo* Ethograms: A Systematic Review and Adaptable Model for Animal Welfare Research

**DOI:** 10.3390/ani16193122

**Published:** 2026-10-05

**Authors:** Débora Silvia Racciatti, Oriol Talló-Parra, Xavier Manteca, Carlos Blanco, Héctor Ricardo Ferrari

**Affiliations:** 1 Cátedra de Bienestar Animal y Etología, Facultad de Ciencias Veterinarias, Universidad de Buenos Aires (UBA), Buenos Aires C1427CWO, Argentina; hferrari@fvet.uba.ar; 2 Animal Welfare Education Center (AWEC), AWEC Advisors S.L., Universitat Autònoma de Barcelona (UAB), 08193 Barcelona, Spain; oriol.tallo@uab.cat (O.T.-P.); xavier.manteca@uab.cat (X.M.); 3 Departamento de Ciencia Animal y de los Alimentos, Facultad de Ciencias Veterinarias, Universitat Autònoma de Barcelona (UAB), 08193 Barcelona, Spain; 4 Cátedra de Sociología Rural, Facultad de Ciencias Veterinarias, Universidad de Buenos Aires (UBA), Buenos Aires C1427CWO, Argentina; cblanco@fvet.uba.ar

**Keywords:** animal welfare, applied ethology, behavioural research, ethogram, lion, systematic review, zoo

## Abstract

Lions (*Panthera leo*) are keystone predators and an iconic species for wildlife conservation. Understanding their behaviour is critical for effective conservation strategies and for ensuring animal welfare in human-managed environments. Ethograms—systematic descriptions of behavioural repertoires—are essential tools for observing, recording, and analysing animal behaviour. Although numerous ethograms have been developed for lions, the lack of standardization and variability in content hinder comparative research and welfare assessment. This study aimed to develop a comprehensive ethogram for lions, based on a systematic review of published sources (following PRISMA guidelines), critical examination of definitions, and video-based observations of captive individuals. As a result, we propose a new ethogram comprising 83 behavioural patterns grouped into 13 functional categories: affiliative (10), aggressive (2), agonistic (7), environment-directed (9), fear-related (2), feeding (9), locomotor (8), maintenance (5), marking (5), potentially stereotypic (7), reproductive (4), resting (7), and other behaviours (8). This ethogram is designed to be both comprehensive and adaptable, supporting its potential application across diverse research contexts, from behavioural monitoring to welfare evaluations. By advancing standardization in behavioural terminology and structure, this work provides a basis for more consistent and comparable data in ethological studies, ultimately supporting evidence-based decision-making in the management and welfare of captive lions.

## Simple Summary

Lions are among the world’s most iconic animals, both for their ecological role and their cultural significance. When lions live in zoos or other facilities under human care, it is essential to ensure their welfare, and one of the best ways to do so is to carefully observe their behaviour. Scientists use a tool called an “ethogram”, a list that describes the different behaviours an animal can show, from resting and feeding to playing or interacting with others. For lions, many ethograms already exist, but they often differ in how behaviours are described and categorized. This makes it difficult to compare studies and apply the knowledge to improve lion care. In this study, we reviewed published research and combined it with video-based observations of lions to develop a unified list of behaviours. The proposed ethogram includes 83 different behaviours grouped into 13 categories and is accompanied by illustrative photographs and annotated video examples. This standardized and adaptable tool is expected to help researchers, zoo professionals, and caretakers better understand lions, improve their welfare, and support conservation and education efforts.

## 1. Introduction

Promoting and continuously improving animal welfare has become a defining challenge for modern zoos [1,2,3,4]. These institutions are no longer viewed solely as exhibition spaces, but as centres of conservation, research, and education, where animal welfare must serve as both a guiding principle and a goal [5,6]. Achieving this requires providing animals under human care with environments and management practices that allow them to express a wide range of natural behaviours [7,8,9]. In this context, large felids—particularly lions (*Panthera leo*)—occupy a central position due to their ecological role as keystone predators [10,11,12], their symbolic and cultural significance [13], and their prominence within zoological collections [7,10,14,15]. According to Species360^®^ Zoological Information Management System (ZIMS), at least 2261 individuals are maintained across 453 facilities [16]. This makes *P. leo* the most common captive felid globally, although these figures are likely underestimated due to incomplete institutional coverage.

Among the available approaches to assess animal welfare, behavioural indicators have been widely employed in different contexts [1,17,18]. Their popularity may stem from their non-invasive data collection [19], lack of reliance on advanced technology [18], and general cost-effectiveness [20]. Beyond welfare assessment, behavioural research also contributes to understanding how animals interact with their physical and social environments, including human–animal relationships, and how these interactions may influence husbandry practices and clinical decision-making [21]. Although behaviour alone does not always provide a complete picture of welfare, it remains a fundamental tool for its assessment [1,18]. Animals may sometimes show normal patterns that mask underlying health problems. However, behavioural indicators can provide valuable information about an animal’s emotional and physical state and may help detect early signs of discomfort, disease, or compromised welfare. This may reveal changes that other measures, such as health indicators, may overlook [22,23].

Ethograms—systematic descriptions of behavioural repertoires—are fundamental for structuring behavioural observations, advancing ethological research, and informing welfare evaluations [20,24,25,26]. They provide standardized and operational definitions of behaviours based on their observable features, including changes in body position, postures, and movements of specific body parts [25]. By breaking the continuous behavioural flow into discrete, observable units, ethograms allow behaviours to be systematically described, categorized, measured, and compared, thereby enhancing objectivity and comparability across studies [21,27,28,29,30]. Ethograms also offer an evidence-based foundation for assessing how well captive environments meet species-specific needs and for designing management practices that support positive welfare outcomes [26,31].

In ethological studies, distinguishing between state behaviours (hereafter “states”) and event behaviours (hereafter “events”) is essential for methodological and measurement purposes [31]. This distinction guides how behaviours are observed, recorded, and quantified, allowing the frequency, duration, and contextual expression to be captured appropriately [31,32,33]. States, such as postures or prolonged interactions, are analysed based on the time spent performing them. Events, in contrast, are brief actions, such as vocalizations or sudden movements, and are quantified by their frequency. This distinction is crucial for selecting the appropriate sampling methods. Instantaneous sampling is used to record states and estimate time budgets, whereas continuous sampling is more effective for events [31].

While numerous ethograms have been developed for lions across various aims and contexts, their partial nature, methodological heterogeneity, and lack of standardisation may lead to inconsistent behavioural recording and reporting, limit cross-study comparability, and constrain the reliable use of behavioural indicators in welfare assessment [21,25,31]. To address this gap, the present study develops a standardized, comprehensive, and adaptable ethogram for lions, based on a systematic review of published sources (following PRISMA guidelines) [34], a critical analysis of behavioural definitions, and video-based observations of captive individuals. This ethogram aims to facilitate more consistent behavioural recording and welfare evaluations, enhance cross-institutional comparisons, and support evidence-based management of *P. leo* in zoological settings.

## 2. Materials and Methods

### 2.1. Systematic Literature Review

**Figure 1:**
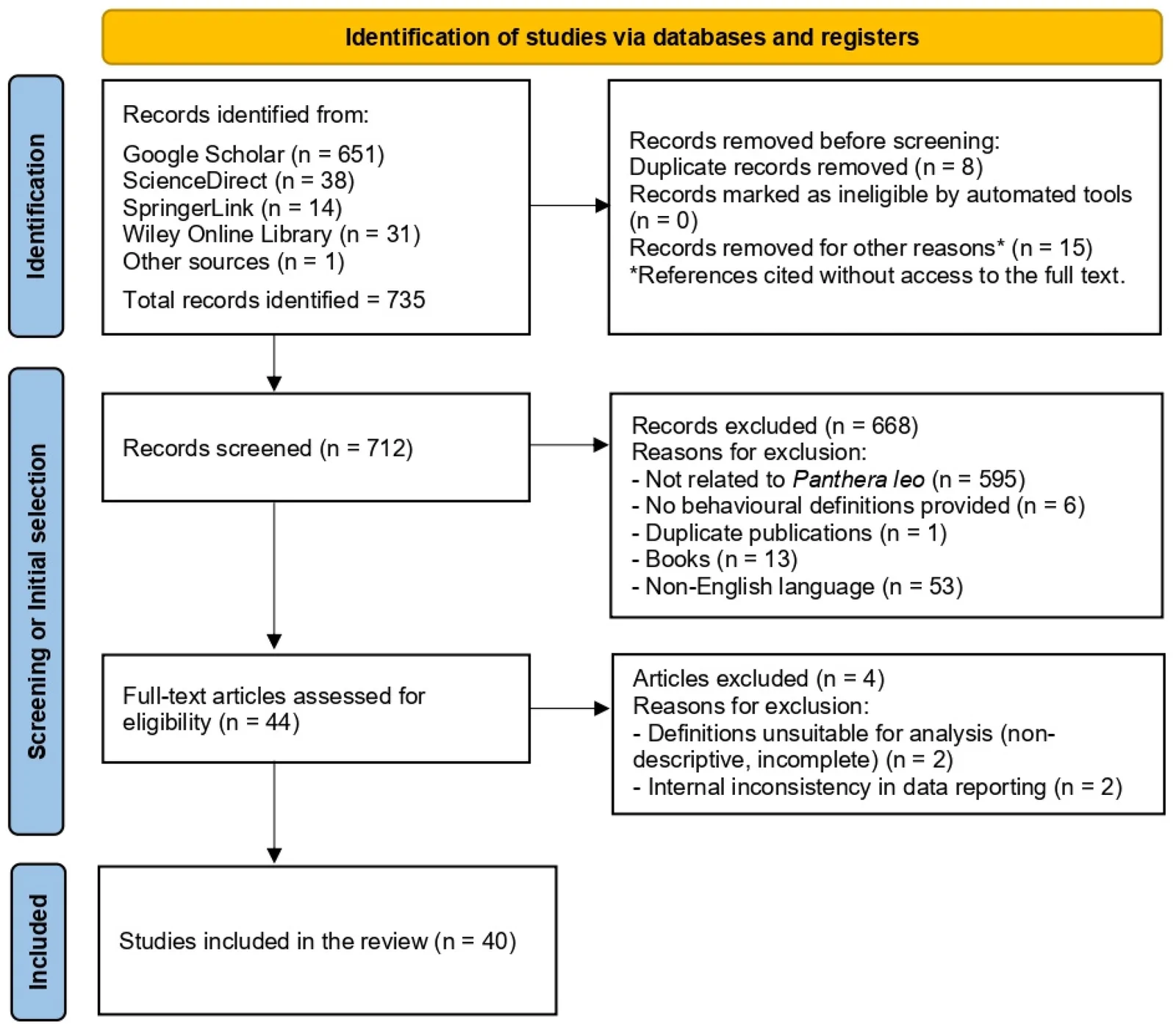
Flow diagram adapted from the PRISMA 2020 model for systematic reviews based exclusively on database and record searches [34]. The process of identification, screening, eligibility assessment, and final inclusion of studies containing relevant *P. leo* ethograms is detailed. * Records removed for other reasons refers to references cited without access to the full text.

We conducted a systematic literature review in accordance with the Preferred Reporting Items for Systematic Reviews and Meta-Analyses (PRISMA) guidelines [34] to ensure transparency and rigor in the identification and selection of relevant studies (Figure 1). The platforms consulted included Google Scholar, ScienceDirect, SpringerLink, Wiley Online Library, and other sources of the grey literature, such as husbandry guidelines for lions (e.g., [35]). We used the search equation “ethogram” AND “*Panthera leo*”, considering publications in English between 1 January 2004 and 31 August 2024.

Inclusion criteria were as follows: (i) the study presented an ethogram or a list of recorded behaviours, either in tabular or textual format; (ii) *P. leo* was the species of interest, either exclusively or in combination with other species; (iii) the publication was a peer-reviewed journal article, a graduate thesis (Master’s or Doctoral), or the relevant grey literature; (iv) the text was written in English.

Studies were excluded if they (i) did not provide descriptive definitions for the listed behaviours; (ii) represented duplicate publications, i.e., multiple publications—typically by the same authors—using identical or non-modified versions of an ethogram, in which case only one version was selected; (iii) were books providing extensive descriptions of species behaviour without an actual ethogram; (iv) could not be accessed through institutional subscriptions, online searches, or direct contact with the authors.

The selection process was conducted in multiple stages (Figure 1). During the screening phase, titles and abstracts were reviewed, and in some cases, full texts were preliminarily accessed to verify basic compliance with the inclusion criteria. At this stage, records were mainly excluded if, although mentioning *P. leo*, the species was not the primary focus of the study, or if the work referenced ethograms without providing a clear list of behaviours and their respective definitions. Additionally, studies in other languages, books, and documents that did not meet the established criteria were removed.

During the eligibility assessment phase, full texts of the selected studies were reviewed to apply the criteria more precisely. Data were collected systematically, recording relevant information from each document, including bibliographic details, study context, and behavioural repertoires and definitions. The level of detail provided in the behavioural descriptions was also assessed, considering whether observable actions were clearly specified and distinguishable from similar behaviours. In addition, whether the ethogram was original, adapted, or directly taken from other authors was recorded. Studies containing behavioural definitions that were non-descriptive or incomplete were excluded from the subsequent definition synthesis, as summarised in Figure 1.

Behavioural definitions extracted from the eligible studies were then systematically reviewed to support their harmonisation and refinement. When multiple definitions were available for the same behaviour, those that minimized ambiguity, avoided tautological (self-referential) formulations or references to presumed adaptive function (i.e., ultimate-level explanations rather than directly observable actions), and provided clear, observable criteria were prioritized. At the same time, contextual or functional information reported in the literature was recorded separately when considered potentially relevant for behavioural interpretation, provided it complemented rather than replaced the observable description of the behaviour. In many cases, complementary elements from different published definitions were harmonised into a single operational definition while preserving the original behavioural meaning. Methodological refinements were made only when necessary to ensure clarity, mutual exclusivity, and consistency with the behavioural patterns observed during the video-based assessment. This process was guided by established methodological recommendations for behavioural description [25,26,27,29,31,36].

### 2.2. Development of a Comprehensive Ethogram for Panthera leo

Following the review of existing ethograms and the refinement of behavioural definitions based on methodological criteria, a new ethogram for *P. leo* was developed. This process involved synthesizing behavioural repertoires reported in the reviewed literature, refining behavioural definitions to enhance operational clarity, and incorporating explicit criteria for body segmentation, posture, and behavioural delimitation. As part of this process, a body segmentation framework was established to standardize anatomical terminology across the reviewed literature and to support the operational definitions of behaviours (Supplementary Figures S1–S3). The aim was to construct an ethogram suitable for applied welfare assessment, addressing the methodological limitations identified in previously published tools while providing transparent criteria that can be adapted according to different research objectives.

Once a preliminary version of the ethogram was established, video observations were conducted by one observer (D.S.R.) to evaluate the applicability of the behavioural definitions and the adequacy of their categorical assignment. This step aimed to determine whether behavioural definitions were clear and operational, and whether behaviours were assigned to mutually exclusive categories appropriate for use in welfare assessment. Given the absence of a gold-standard ethogram for *P. leo*, completeness was assessed by integrating all behavioural categories reported in the reviewed literature—adapted to ensure mutual exclusivity—and by confirming through video observations that all observed behaviours could be described using the proposed definitions. A total of 8 h 46 min and 46 s of audiovisual material was analysed. Of this, 44 min and 5 s corresponded to recordings collected at the Barcelona Zoo (Spain), 2 h 18 min and 55 s at the Madrid Zoo Aquarium (Spain), and 2 h 44 min and 11 s at Parco Faunistico Le Cornelle (Bergamo, Italy); these recordings were obtained during routine management contexts by the first author and by external collaborators who contributed footage. An additional 2 h 59 min and 35 s consisted of videos sourced from publicly available YouTube^®^ uploads. Online and collaborator-provided videos were selected using explicit criteria: (i) clear and continuous visualization of lions suitable for behavioural scoring; (ii) minimal or no editing that could bias interpretation; and (iii) sufficient image resolution to identify postures and body-part positions.

To support the development of operational definitions, behavioural patterns were characterised according to temporal criteria. The sampling interval was used as a reference for proposing a state/event classification. Behaviours whose observed duration was less than 1 min—i.e., shorter than the pre-established sampling interval—were considered events. Some behaviours exhibited variable durations, allowing their classification as either states or events according to the operational criteria established by the authors, in line with [7]. For behaviours that were not directly observed, recorded on video, or identified in the reviewed YouTube^®^ footage (see Supplementary Material Lion Ethogram S4), the proposed state or event classification was informed by criteria reported in the reviewed literature. When no explicit guidance was available, classification was based on the expected duration of the behaviour, adopting a conservative, case-by-case approach.

To support the operational description of behaviours considered events within the proposed classification, criteria were established to define when a behaviour should be considered a new occurrence. These criteria were organised into three dimensions: temporal, spatial, and postural. The temporal dimension focused on the interval between actions or behavioural changes; for example, for “lick”, a new occurrence was recorded when there was a pause of more than 5 s between one series of licks and the next. This approach was adapted from the “5-s rule” proposed by Powell and Baskir [37], originally described for state behaviours, and applied here to event recording to avoid artificially fragmenting a continuous motor sequence into multiple occurrences. The spatial dimension considered distances between individuals and changes in their relative position; for example, for “social approach”, a new occurrence was recorded each time an individual approached another to a distance shorter than two body lengths. This criterion was adapted from Boyle et al. [38] as a standardized operational measure of proximity, providing an explicit operational rule for determining when a new behavioural occurrence should be recorded. The postural dimension referred to variations in body position that signalled transition between behaviours. For example, in “rear up”, a new occurrence was recorded only after the animal returned to a four-limb stance before rearing again.

## 3. Results

### 3.1. Systematic Literature Review

The primary search yielded a total of 735 results, which were subjected to screening and eligibility assessment. After this process, 40 studies were selected for the final analysis (Table 1). Most studies (92.5%, *n* = 37) were conducted in captive settings; one was carried out in the wild, another compared both conditions (captive vs. wild), and one consisted of a review of preexisting studies. Except for this latter study, which proposed a standardized ethogram based on a review of domestic and wild felids, most studies (97.5%, *n* = 39) used ethograms as methodological tools to address specific research questions rather than as primary study outcomes (Table 1).

None of the reviewed studies explicitly defined the anatomical terminology considered when defining behaviours or provided complementary visual resources (illustrations, videos, or audio recordings).

Regarding behavioural categories, 77.5% (*n* = 31) of studies reported them clearly, while 22.5% (*n* = 9) either did not categorize behaviours or provided unclear information. The number of categories ranged from 2 to 15 (mean = 5.6; median = 5; mode = 3), with only three studies presenting more than 10 categories. Grouping criteria varied, from general categories (e.g., activity, inactivity, environmental enrichment) to more detailed or functional classifications. The number of behaviours per ethogram ranged from 4 to 107 (mean = 23.35; median = 18; SD = 18.1), reflecting high variability.

The quality and clarity of behavioural definitions were inconsistent across studies. Ambiguity was identified in 75% of studies (*n* = 30), tautological definitions in 72.5% (*n* = 29), and lack of operational criteria in 15% (*n* = 6). Moreover, 67.5% of studies (*n* = 27) included references to presumed function or behavioural outcomes (e.g., why the behaviour occurs or what it achieves, rather than how it is expressed), which obscured the observable form of the behaviour rather than complementing it. Similarly, 52.5% (*n* = 21) incorporated elements related to internal states or intentionality. Overlap between categories due to unclear exclusion criteria was observed in 27.5% of studies (*n* = 11). Additionally, only 22.5% of studies (*n* = 9) explicitly reported a proposed state/event classification for the behavioural patterns included in the ethogram.

These findings informed the development of the comprehensive ethogram presented in this study, with particular emphasis on operational clarity, mutual exclusivity, and standardisation of behavioural definitions.

### 3.2. Comprehensive Ethogram for Panthera leo

The main outcome of this study was the development of a comprehensive ethogram for *P. leo,* comprising 83 behavioural patterns organised into 13 functional categories: affiliative (10), aggressive (2), agonistic (7), environment-directed (9), fear-related (2), feeding (9), locomotor (8), maintenance (5), marking (5), potentially stereotypic (7), reproductive (4), resting (7), and other behaviours (8). The complete ethogram, including detailed operational definitions of behaviours, illustrative images, and annotated video examples, is provided as Supplementary Materials (Lion Ethogram S4). Due to space constraints, only a concise overview of the behavioural categories and patterns, together with their corresponding general welfare associations, is presented in Table 2. These literature-informed annotations are provided as complementary information for readers interested in welfare assessment. They should not be interpreted as fixed welfare values or intrinsic properties of the behaviours, as the welfare significance of any behaviour depends on its context, intensity, duration, and consequences.

### 3.3. Conceptual Representation of Behavioural Resolution Levels for Ethogram Application

**Figure 2:**
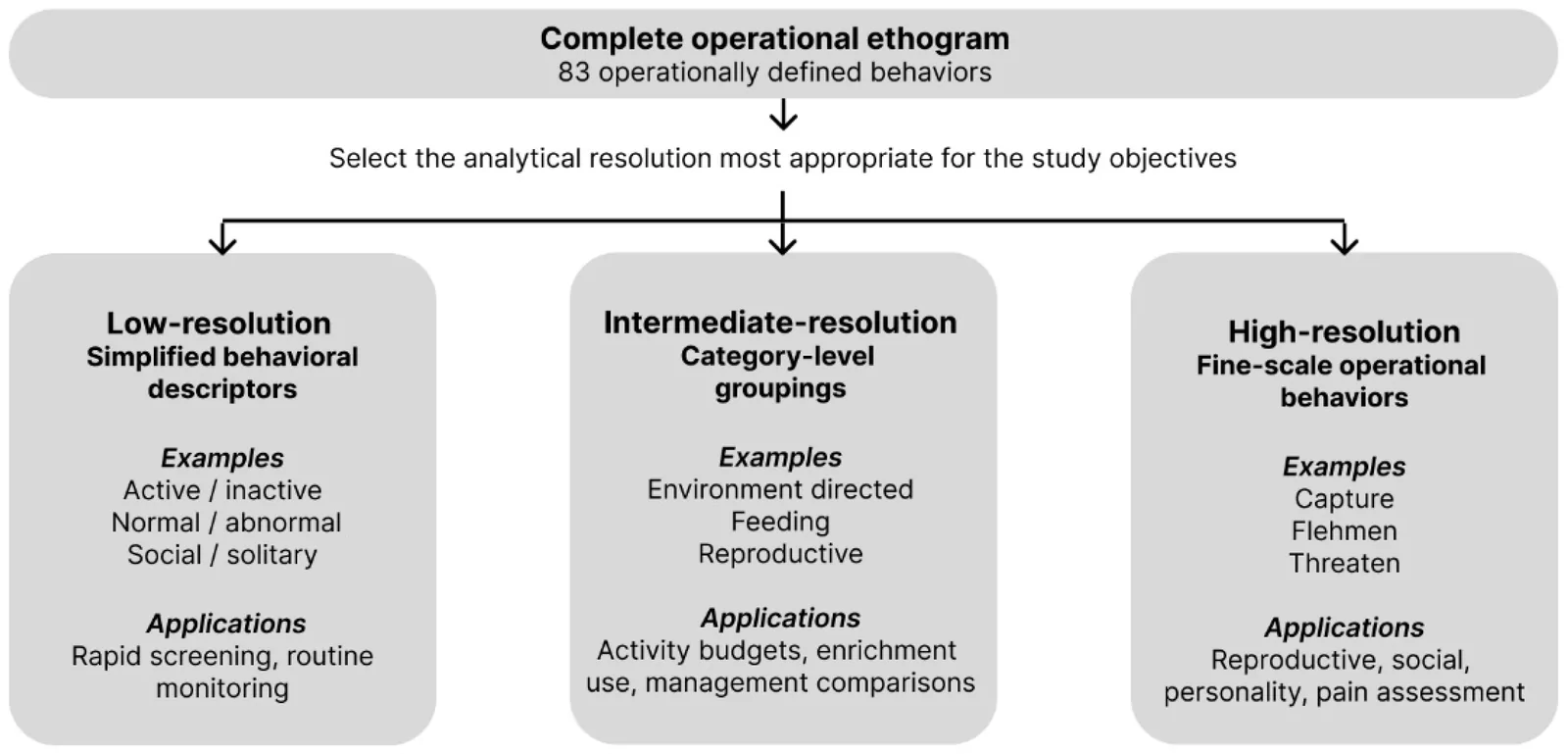
Conceptual representation illustrating examples of selectable analytical resolution for study-specific application of the lion ethogram. The complete operational repertoire (83 behaviours) serves as a common reference from which researchers may select simplified behavioural descriptors, category-level groupings, or fine-scale operational behaviours according to their study objectives, required sensitivity, and observational constraints. Examples shown at each level are illustrative rather than exhaustive.

This section presents a conceptual representation of behavioural resolution levels to facilitate the practical application of the complete lion ethogram across studies with different objectives and observational requirements. As illustrated in Figure 2, the full operational repertoire may serve as a common reference from which researchers can selectively use different levels of behavioural resolution according to the specific aims of a study, the sensitivity required, and the feasibility of data collection.

Rather than representing a fixed classification system, the conceptual representation illustrates alternative and non-mutually exclusive analytical resolutions that may be selected during study design according to study objectives, required sensitivity, and observational constraints. These include simplified behavioural descriptors for rapid screening or routine monitoring, category-level groupings for activity budgets or management-oriented assessments, and fine-scale operational behaviours for detailed behavioural, social, reproductive, or pain assessment studies.

This structure is intended as a flexible reference tool that preserves the completeness of the ethogram while facilitating its adaptation to different research contexts. Importantly, the representation does not imply that all behaviours must be observed in every study, but rather that researchers may select the subset and level of behavioural detail most relevant to their specific research questions and monitoring capacities.

## 4. Discussion

### 4.1. Methodological Gaps Identified in Published Panthera leo Ethograms

The predominance of captive-based studies, consistent with Stanton et al. [44], likely reflects the practical advantages of these environments, including accessibility, control of variables, and facilitated systematic data collection [69,70]. However, behaviour observed under such conditions may not fully reflect the behavioural repertoire expressed under natural ecological conditions [44,71], which should be considered when interpreting behavioural data. The limited number of studies conducted in the wild or under comparative conditions further highlights the need to better understand how ecological context influences behavioural indicators used in animal welfare research.

Across the reviewed literature, ethogram construction was generally treated as a supporting methodological component rather than as an object of systematic development. Combined with the space limitations inherent to scientific journals, this often resulted in simplified behavioural inventories that remain difficult to compare or reuse across studies [44].

The lack of explicit anatomical references, body segmentation, and supporting visual material further limited the operational clarity of behavioural definitions, despite recommendations that behavioural descriptions should be sufficiently objective and detailed to ensure consistent recognition by different observers [25,26]. These findings suggest that greater attention to the methodological development and documentation of ethograms could substantially improve their transparency and facilitate more consistent behavioural recording, particularly in animal welfare research, where behavioural observations constitute a central assessment tool [1,62,72].

The review also revealed considerable heterogeneity in the number, structure, and organisation of behavioural categories. Rather than indicating methodological shortcomings, this variability largely reflects differences in research objectives and observational requirements. Studies aimed at rapid behavioural screening or routine monitoring may legitimately adopt simplified behavioural repertoires, whereas investigations focusing on social behaviour, pain assessment, or behavioural diversity often require greater behavioural resolution. Consequently, no single level of behavioural categorisation is universally appropriate. Instead, behavioural resolution should be selected according to the research question while maintaining sufficient internal consistency to facilitate interpretation and, where possible, comparisons across studies [25,73,74,75].

Building on the importance of clear behavioural definitions, the distinction between states and events constitutes another critical methodological decision in ethogram design. Although this distinction is well established in the literature [25,75,76], its application is not always reported explicitly, which may reduce transparency and hinder comparisons across studies. Clear classification criteria are particularly relevant for welfare-oriented research, where the choice between recording behaviours as states or events can influence behavioural quantification and subsequent interpretation [32,37,77]. In the present ethogram, the proposed classification of behaviours as states or events was developed from behavioural observations, information extracted from the reviewed literature, duration-based criteria, and explicit operational definitions for context-dependent behaviours, as detailed in Section 2.2.

### 4.2. Methodological Principles Underlying the Development of the Ethogram

#### 4.2.1. Supporting Operational Clarity Through Anatomical References and Visual Resources

A central methodological feature of the proposed ethogram is the integration of anatomical references with illustrative images and annotated videos to support behavioural identification. Although operational definitions remain the primary basis for behavioural recording, visual resources can facilitate their interpretation, particularly when behaviours differ through subtle postural or expressive variations [25]. As noted by Lahitte et al. [25], verbal descriptions alone may not always ensure consistent interpretation across observers, and complementary visual materials may facilitate observer training and behavioural coding. The anatomical references included in the ethogram are not intended to define fixed external boundaries, but rather to provide a common descriptive framework for identifying the body regions involved in each behaviour [25]. Similarly, the accompanying images and videos are intended to illustrate the operational definitions rather than replace them. Together, these resources may improve methodological transparency and facilitate the consistent application of behavioural definitions across studies [25,76,77].

#### 4.2.2. Classification Decisions and Methodological Trade-Offs

One of the principal challenges in developing a comprehensive ethogram lies in balancing behavioural resolution with classificatory consistency. Decisions regarding whether behaviours should be separated, grouped, or assigned to particular functional categories inevitably involve methodological trade-offs [25,29]. In the present ethogram, priority was given to operational definitions based on observable form, mutually exclusive categories, behavioural resolution, and transparency regarding the rationale underlying each classificatory decision [25,29,31]. Because different research questions require different levels of behavioural detail, these decisions should be regarded as methodological choices rather than universally applicable solutions. An illustrative example is the classification of “social resting”, defined as situations in which an animal is in any resting posture while maintaining physical contact with one or more conspecifics, but without active social interaction (Supplementary Materials Lion Ethogram S4). Two approaches were identified in the reviewed literature, with “social resting” classified as either social (e.g., [7,12,44]) or resting/inactive (e.g., [41,44]). Because social resting simultaneously reflects energy conservation and affiliative contact, neither interpretation is inherently incorrect. In the present ethogram, it was assigned to the resting category to maintain mutually exclusive classifications and facilitate activity-budget analyses [25,29,32,33]. However, in studies focusing on social relationships or affective welfare, the same behaviour may equally be considered an affiliative indicator. This example illustrates that behavioural classification should ultimately be guided by study objectives while remaining transparent and internally consistent. Accordingly, posture-based resting states were retained as separate behavioural entries, whereas alert and social resting were defined by attentional or social characteristics while referring to the relevant resting posture.

Similar considerations guided the distinction between “Sniff” and “Explore”. Although both behaviours involve olfactory investigation, they were retained as separate behaviours because they differ in their locomotor component and, consequently, in the type of information their distinction provides for behavioural analyses. “Sniff” describes stationary olfactory investigation, whereas “Explore” refers to olfactory investigation performed during locomotion. This distinction allows researchers to differentiate between stationary investigation of a specific stimulus and broader environmental exploration, as well as between lower- and higher-activity forms of environmental investigation. Such information may be particularly relevant in welfare assessments, activity-budget analyses, and studies evaluating responses to environmental enrichment [13,78,79,80]. Nevertheless, researchers interested primarily in exploratory motivation may readily combine both behaviours into a broader exploration category according to the objectives of their study.

A related challenge concerns behaviours that share the same observable motor pattern but occur in different contexts. Whenever possible, behaviours in the present ethogram were defined according to their observable form, while contextual information was retained only as complementary information for interpretation. However, when both the systematic review and our observational material supported distinct contextual expressions, separate entries were retained to preserve analytical resolution without modifying the operational description of the behaviour. “Charge” and “Stalk” illustrate this principle. Although both behaviours may theoretically occur in different contexts, all published descriptions identified in the reviewed literature classified “Charge” exclusively within aggressive interactions [8,13,44], and this behaviour was not observed outside aggressive contexts in our video material. Accordingly, “Charge” was retained as an aggressive behaviour. In contrast, stalking was reported across multiple contexts. To avoid unsupported functional assumptions while maintaining mutually exclusive categories, “Stalk” was subdivided according to the target involved (prey, object or individual), whereas the operational description of the motor pattern remained unchanged. This approach preserves behavioural resolution while allowing researchers to use broader behavioural categories when appropriate.

Potentially stereotypic behaviours represent a particularly important challenge in ethograms intended for animal welfare assessment, as their identification may have important implications for welfare interpretation [26,32]. In this study, behaviours such as “sniff” and “stereotypic sniffing” were differentiated based on repetition and pattern. However, this distinction is methodologically complex, as stereotypies are often defined by rigid repetition and apparent lack of function [25,26,81], making the boundary between normal and potentially stereotypic behaviour difficult to establish using observable criteria alone [25,32]. Moreover, establishing a minimum number of repetitions to define a behaviour as stereotypic is inherently arbitrary and context-dependent [26]. In the reviewed literature, only 10 out of 40 ethograms explicitly reported a minimum repetition threshold. Six studies used three repetitions [11,47,58,60,61,67], while three adopted a two-repetition criterion [39,45,56]. Given this variability, a criterion of three consecutive repetitions was adopted in the present study as a pragmatic field-based threshold, while acknowledging its provisional and context-dependent nature. Additionally, to avoid overinterpretation, these behaviours were categorised as “potentially” stereotypic behaviours, emphasising the need for further assessment based on behavioural and physical consequences (e.g., injuries, weight loss) and environmental context [82].

Finally, several behaviours included in the ethogram were not directly observed but were incorporated from previous publications because they represent relevant components of the behavioural repertoire. These entries were explicitly identified and should be regarded as provisional until additional empirical observations become available. Including them preserves the completeness of the ethogram while maintaining transparency regarding the level of empirical support available for each behavioural definition.

### 4.3. Behavioural Resolution and Welfare Interpretation in the Application of the Lion Ethogram

The need for considering different levels of behavioural resolution became evident during the systematic review, which revealed substantial heterogeneity among published lion ethograms regarding behavioural terminology, repertoire size, and descriptive depth. By integrating these dispersed descriptions into a comprehensive operational repertoire, the present ethogram provides a reference resource that can support different research applications. Different levels of behavioural resolution may be appropriate for different research questions and management purposes [44,60,83,84]. Simplified behavioural descriptors may be sufficient for rapid behavioural screening and routine welfare monitoring [37,38,75,78], whereas category-level groupings may better support activity-budget analyses, environmental enrichment studies, and management comparisons [9,10,80,85]. Fine-scale operational behaviours may be required for questions involving reproductive behaviour, social interactions, personality assessment, or subtle behavioural indicators such as pain-related responses [32,65,80,84]. Rather than representing alternative ethograms, these levels illustrate different ways of applying the same operational repertoire according to the objectives, analytical sensitivity, and practical constraints of each study.

Although Figure 2 focuses on behavioural resolution, Table 2 complements this conceptual representation by summarising the general welfare associations reported in the literature for the behavioural categories included in the ethogram. These associations are intended to complement, rather than replace, behavioural interpretation and provide a general guide based on published evidence, recognising that the welfare significance of any individual behaviour depends on its context [1,86,87], as well as its intensity, duration, frequency [1,88], and consequences for the individual [82,89]. This aligns with conceptual frameworks such as the Five Domains Model, which emphasise that observable behavioural expressions gain welfare relevance through their relationship with internal affective experiences shaped by context and environment [90].

Behaviours such as play, affiliative interactions, and exploratory activities are frequently interpreted as indicators of positive welfare, as they typically occur only when basic needs are met and when environmental conditions are favourable [1,91,92,93]. Furthermore, while social interactions buffer stress and strengthen group cohesion, motivated exploration and play serve as windows into positive affective states and the experience of pleasure [1]. Conversely, stereotypic behaviours, persistent fear responses, or unmitigated aggression are commonly associated with compromised welfare, particularly when they are repetitive, inflexible, or disproportionate to the immediate context [1,89,93].

Nonetheless, many behaviours remain inherently context-dependent. Yawning, for example, in different species has been observed to be associated with stress, fatigue, or boredom [94], but it also constitutes a normal physiological response related to thermoregulation and brain oxygenation [95,96]. In social settings, as observed in multi-cat households, it may further function as a calming or affiliative signal, contributing to tension reduction rather than indicating negative welfare per se [97]. In lions, yawning has been specifically associated with a state of rest [11,41,44], reduced vigilance [9,41], or post-feeding recovery [10].

Similarly, scent marking is not intrinsically problematic in territorial species such as lions [12,41,50,98]; however, when expressed excessively or compulsively in captive environments, it may reflect stress, frustration, or insufficient stimulation [99]. In such cases, the same behaviour may shift from a normal territorial response to a potential indicator of compromised welfare, underscoring the need for context-sensitive interpretation.

Overall, the integration of behavioural resolution considerations with contextual interpretation of welfare relevance highlights the importance of distinguishing behavioural description from behavioural meaning. A comprehensive ethogram can support consistent behavioural recording, but the interpretation of its welfare implications requires consideration of the biological function of each behaviour and the circumstances in which it is expressed.

### 4.4. Limitations and Future Directions

Although the present ethogram represents the most comprehensive operational repertoire currently available for *P. leo* based on the evidence reviewed, it should not be regarded as a definitive or exhaustive catalogue. Behavioural repertoires remain inherently open to refinement as new behavioural patterns are documented under different ecological, management, or experimental conditions [25,29].

The restriction of the systematic review to publications written in English represents a potential limitation, as relevant behavioural descriptions published in other languages may not have been considered. Future reviews incorporating multilingual assessment strategies may further expand the available evidence based on lions’ behavioural repertoires.

Some behavioural definitions were necessarily adapted from published sources because they could not be directly observed in our own material. Although these behaviours were retained to support the completeness of the ethogram, future empirical observations may allow further refinement of their operational definitions.

Future studies should evaluate the applicability, reliability, and reproducibility of the ethogram across different observers, institutions, captive and free-ranging populations, and observational contexts. Such validation will help determine its generalisability and inform its broader use in behavioural and welfare research.

In addition to methodological validation, future research should also investigate the welfare significance of individual behavioural patterns and their combinations through empirical assessment, thereby strengthening the links between operational behavioural recording and evidence-based animal welfare assessment. As behavioural evidence continues to accumulate, the present ethogram should be regarded as a dynamic reference tool that can be progressively refined and expanded to support increasingly robust behavioural and welfare investigations.

## 5. Conclusions

The literature review conducted in this study confirms the considerable methodological heterogeneity that characterises published lion ethograms, particularly regarding behavioural definitions, category structure, and reporting practices. Such variability limits comparability among studies and hinders the cumulative development of behavioural knowledge, highlighting the need for greater methodological transparency in ethogram construction and reporting.

To address these challenges, we developed a comprehensive operational ethogram for *P. leo* based on a systematic review of the literature, critical methodological evaluation, and preliminary evaluation using behavioural observations and video records. The resulting ethogram incorporates explicit operational definitions, anatomical references, visual resources, and transparent classification criteria designed to support consistent behavioural recording and facilitate its application across different research contexts. Importantly, it was developed with an applied animal welfare perspective, providing a structured behavioural reference tool that can support welfare assessment and monitoring by facilitating the identification and interpretation of behavioural indicators. However, this resource should be considered a methodological aid rather than a definitive assessment tool, as its inter-observer reliability, generalisability, and welfare relevance require further empirical evaluation and integration with additional welfare measures.

Rather than proposing a fixed behavioural classification system, the conceptual representation presented in this study illustrates how the same operational repertoire may be applied at different levels of behavioural resolution according to study objectives and analytical requirements. This flexible approach allows researchers to adapt the ethogram to diverse applications while maintaining a common methodological reference that facilitates comparisons among studies.

Although developed specifically for *P. leo*, the methodological approach adopted here may also provide a useful reference for developing comprehensive ethograms for other species. Future research should continue evaluating the proposed behavioural definitions across different populations and contexts and formally assess inter-observer reliability, while strengthening the evidence linking behavioural indicators with animal welfare outcomes. This will support increasingly robust, standardised, and evidence-based behavioural assessment and improve the application of behavioural monitoring in animal welfare management.

## Figures and Tables

**Table 1:** Selected studies from the systematic literature review of ethograms for *Panthera leo*, indicating study context and the research objectives supported by the ethogram. Studies are presented chronologically according to publication year to reflect the temporal evolution of ethogram use in *P. leo* research. (*) Review study based on the previously published literature; (**) study conducted in the wild; (***) study conducted in both captive and wild settings. Unmarked studies were conducted in captive settings. Abbreviations used in the table are as follows: AW, animal welfare; EE, environmental enrichment; SB, stereotypic behaviour.

Study Reference	Ethogram Use
Bashaw et al. [39]	Activity patterns, AW, and SB under different housing conditions.
Umapathy et al. [40]	Oestrous behaviour monitoring.
Van Metter et al. [9]	EE effectiveness and inter-individual behavioural differences.
Lyniuk [41]	Activity budgets and space use.
Szokalski et al. [42]	Effects of visitor interactions on AW.
Martínez-Macipe et al. [43]	Effects of olfactory EE.
Stanton et al. [44] *	Comparative behavioural studies of *Felidae*.
Dunston et al. [45] **	Behavioural phenotypes, sociality, and boldness.
Stryker [46]	Thermoregulatory behaviour and space use.
Kohari et al. [47]	Activity budgets and behavioural motivation.
Pastorino et al. [48]	Personality-related behavioural responses to novel environments.
Schildkraut [7] ***	Behavioural budget comparisons between captive and wild populations.
Suárez et al. [49]	Effects of visitor presence on behavioural patterns and space use.
Cederlund [50]	Behavioural responses to olfactory stimuli.
Iske et al. [51]	Effects of pig head EE as a biologically relevant stimulus.
Khan et al. [52]	Relationship between available space and behavioural expression.
Sgambelluri et al. [53]	Behavioural responses during evaluation of a non-invasive saliva collection device.
van Dam [54]	Effects of humans as visual stressors.
Höttges et al. [10]	Effects of feeding frequency on behaviour and activity.
Moloney [11]	Behavioural responses in relation to individual and environmental variables.
Pelletier [55]	Effects of noise and visitor presence on AW.
Regaiolli et al. [56]	Effects of visual motion illusions on AW.
Boyle et al. [38]	Visitor effects and inter-individual behavioural variation.
Finch et al. [57]	Effects of carcass feeding on activity budget.
Goswami et al. [58]	Effects of rearing history and personality on behavioural welfare.
Jansen & Thodberg [59]	Behavioural comparison between female lions with and without contraceptive implants.
Siahaan et al. [14]	Daily activity patterns and SB.
Babb [60]	Felid behaviour in zoos and sanctuaries.
Galardi et al. [12]	Effects of group housing on behaviour, sociability, and enclosure use.
Goswami et al. [13]	Effects of combined EE on AW.
Williams et al. [61]	AW-related behavioural responses and inter-individual variation.
Seyrling et al. [62]	Effects of environmental and husbandry factors on behavioural budgets.
Amundrød [63]	Effects of feeding regimes on daily activity budgets, SB, and enclosure use.
Goswami et al. [8]	Effects of visitor presence and enclosure complexity on AW.
Wilson & Phillips [64]	Behavioural responses of cubs to wildlife tourism and human interaction frequency.
Ali et al. [15]	Seasonal effects on captive carnivore behaviour.
Callealta et al. [65]	Hormonal variation and reproductive behaviour patterns.
Ahsan et al. [66]	Effect of essential oils on stress coping.
Fitskie et al. [67]	Effects of a random feeding schedule on pacing behaviour.
Salma et al. [68]	Sound levels and associated behavioural patterns.

**Table 2:** Overview of behavioural categories and patterns included in the *P. leo* ethogram. Behavioural categories and their corresponding patterns are organized in alphabetical order. EV, behavioural event; ST, behavioural state. The “General welfare association” column summarises broad associations between individual behaviours and animal welfare described in previous studies and is provided as contextual information only: (+), generally associated with positive welfare states; (−), generally associated with negative welfare states; (+/−), context-dependent. These associations do not represent fixed properties of behaviours; rather, their welfare significance depends on the context in which they are expressed, as well as their intensity, duration, frequency, and consequences for the individual.

Category	Behaviour	Proposed Recording Type	General Welfare Association
**Affiliative behaviours** Behaviours in which the lion is close to another individual (no more than two body lengths apart) and performs actions associated with bond formation.	Allogroom	EV or ST	(+)
	Follow	EV or ST	
	Head rub	EV	
	Head nudge	EV	
	Nurse	EV or ST	
	Nuzzle	EV	
	Receive	EV or ST	
	Social approach	EV	
	Social play	EV or ST	
	Social sniff	EV	
**Aggressive behaviours** Behaviours in which the lion is close to another individual (no more than two body lengths apart) and performs actions associated with conflict or competition involving physical contact.	Fight	EV or ST	(−)
	Tackle	EV	
**Agonistic behaviours**	Displace	EV	(+/−)
	Flatten		
Behaviours in which the lion is close to another individual (no more than two body lengths apart) and displays actions associated with conflict or competition, without physical contact.	Retreat		
	Social flee		
	Social pursue		
	Submit		
	Threaten		
**Environment-directed behaviours**	Chew object	EV or ST	(+/−)
	Dig	EV	
Behaviours in which the individual investigates the surroundings or a specific stimulus. This category includes behaviours directed toward environmental enrichment. It does not include marking behaviours.	Explore	EV or ST	
	Flehmen	EV	
	Lick	EV	
	Manipulate	EV or ST	
	Stalk object	EV or ST	
	Sniff	EV	
	Rear up	EV or ST	
**Fear-related behaviours**	Flee	EV	(−)
Behaviours characterized by avoidance responses, immobility, and heightened vigilance, commonly associated with situations involving actual or perceived threats or environmental disturbances.	Freeze	EV or ST	
**Feeding behaviours**	Attack prey	EV	(+/−)
	Capture	EV	
Behaviours associated with the acquisition and ingestion of food or water, including feeding enrichment.	Chase prey	EV	
	Chew food	EV or ST	
	Drink	EV	
	Kill	EV	
	Stalk prey	EV or ST	
	Suckle	EV or ST	
	Transport food	EV	
**Locomotor behaviours**	Ascend	EV	(+/−)
	Climb	EV	
Behaviours that enable movement from one place to another.	Descend	EV	
	Jump	EV	
	Run	EV	
	Swim	EV	
	Trot	EV	
	Walk	EV or ST	
**Maintenance behaviours**	Self-scratch	EV	(+/−)
	Self-groom	EV or ST	
Behaviours associated with physiological needs and self-care, excluding “Feeding behaviours” and certain “Marking behaviours”.	Stretch	EV	
	Urinate	EV	
	Yawn	EV	
**Marking behaviours**	Defecate	EV	(+/−)
	Rub object		
Behaviours related to communication between individuals, through which olfactory or visual signals are deposited on a surface, generally for territorial, reproductive, or social purposes.	Scrape with hind paws		
	Scratch		
	Spray		
**Not observable:** This is not a category or behaviour, but an annotation that must be recorded when the focal animal is not visible, or is only partially visible, making it impossible to accurately determine which behaviour it is performing.			
**Other behaviours**	Air-bite	EV	(+/−)
	Crouch	EV or ST	
Behaviours that do not fit into any of the defined categories. This category includes actions that cannot be reliably classified elsewhere due to their ambiguity, rarity, or context-dependent nature.	Head-shake	EV	
	Lip-lick	EV	
	Mount	EV or ST	
	Stalk	EV or ST	
	Stand	ST	
	Vocalize	EV	
**Potentially stereotypic behaviours**	Head-move	EV or ST	(−)
	Forepaw lift		
Repetitive behavioural patterns with low or no variability. The behaviour must be performed three or more times consecutively for it to be considered stereotypic.	Pace		
	Self-mutilate		
	Stp. muzzle rub		
	Stp. Rub		
	Sway		
**Reproductive behaviours**	Copulation	EV	
	Post-copulatory anogenital grooming	EV or ST	(+)
Sexual behaviours associated with mating and reproduction.	Post-copulatory rolling	EV	
	Reproductive evasion	EV or ST	
**Resting behaviours**	Alert resting	ST	(+/−)
	Dorsal recumbency		
ST in which the individual remains immobile or with minimal movement, without displacement. When, during resting, the individual simultaneously performs a behaviour belonging to another category (e.g., “milk”), the behaviour is recorded under that category and not as resting.	Lateral recumbency		
	Perching		
	Sitting		
	Social resting		
	Ventral recumbency		
	Alert resting		

## Data Availability

The behavioural ethogram and the edited video clips supporting the findings of this study are available in the Supplementary Materials. Raw video data are not publicly available due to ethical restrictions related to the use of recordings of animals under human care. However, the data may be available from the corresponding author upon reasonable request.

## Supplementary Materials

The following supporting information can be downloaded at https://www.mdpi.com/article/10.3390/ani16193122/s1.
