# Supplementary material for: Bridging Gaps in Panthera leo Ethograms: A Systematic Review and Adaptable Model for Animal Welfare Research
Source: Animals (Basel). 2026 Oct 5;16(19):3122. doi: 10.3390/ani16193122 (PMC13663599; doi:10.3390/ani16193122)
Supplement: Supplementary file 1 [file animals-16-03122-s001.zip › Figure_S1_BodySegmentation.pdf]

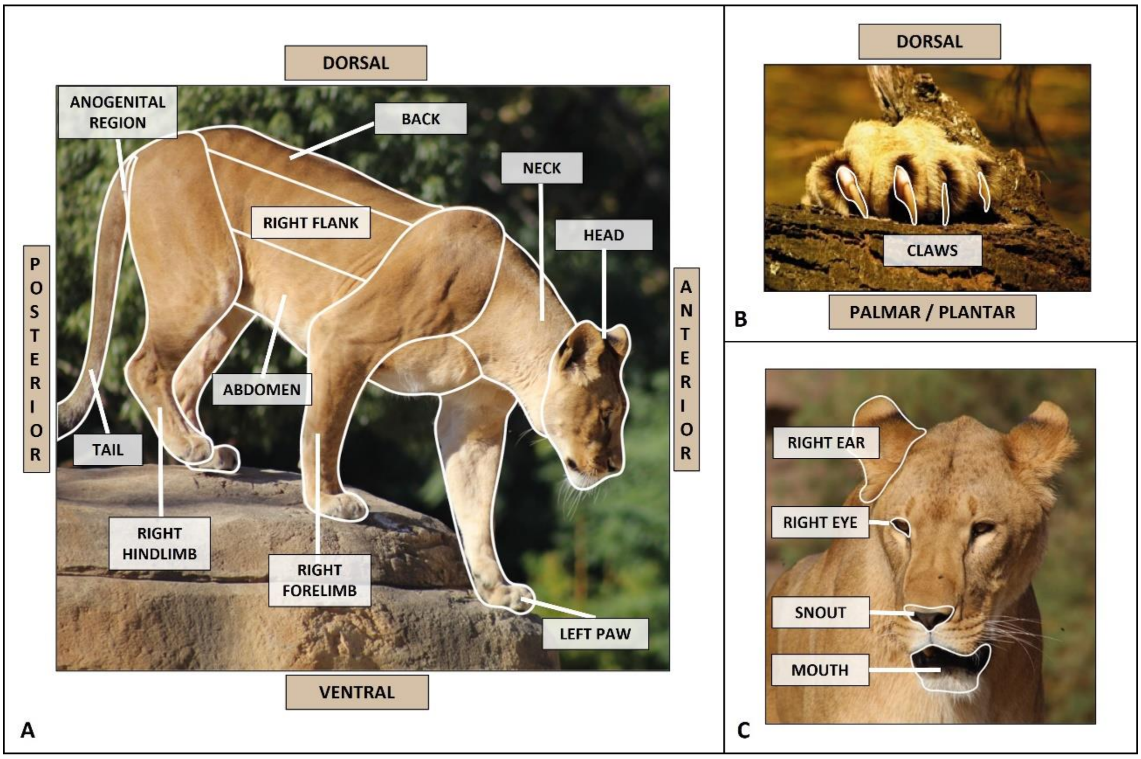

Figure S1. Diagram of the body segmentation of a lioness. The main anatomical regions used in the ethogram definitions are indicated. A: Right lateral view of the whole body. B: Detail of a paw resting on a log, where the extended claws can be observed. C: Detail of the head and its components. Credits: photos A and C by Laura Zarraoa, photo B by David Youldon (Dreamstime.com).
