# Supplementary material for: Bridging Gaps in Panthera leo Ethograms: A Systematic Review and Adaptable Model for Animal Welfare Research
Source: Animals (Basel). 2026 Oct 5;16(19):3122. doi: 10.3390/ani16193122 (PMC13663599; doi:10.3390/ani16193122)
Supplement: Supplementary file 1 [file animals-16-03122-s001.zip › Figure_S2_Positions_MainAnatomicalRegions_Head.pdf]

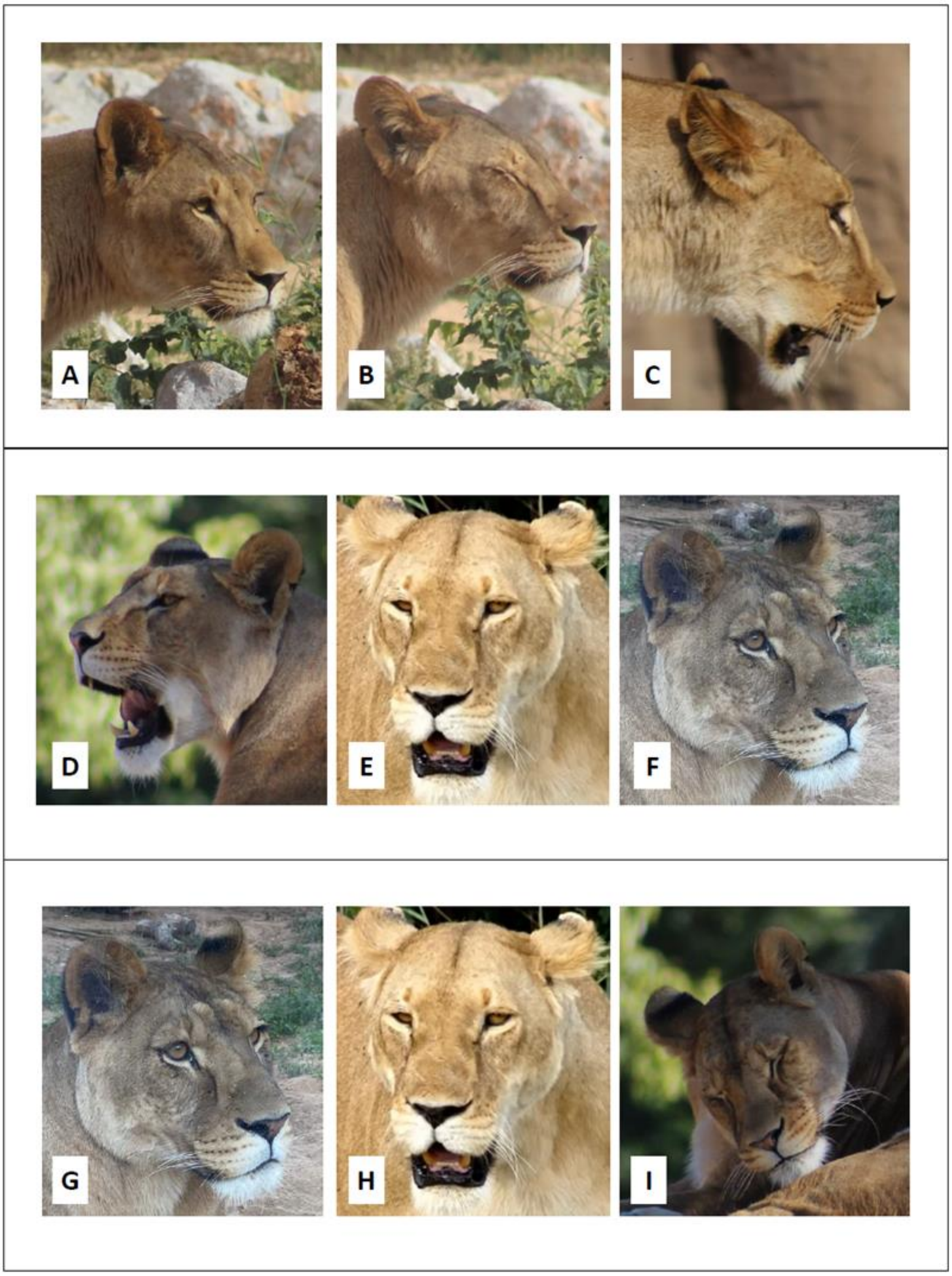

Figure S2. Positions that the main anatomical regions of the head can adopt, as used in the *P. leo* ethogram definitions. Top: the ears may be forward (A), backward (B), or flattened (C). Middle: the mouth may be open (D), partially open (E), or closed (F). Bottom: the eyes may be open (G), half-open (H), or closed (I). Credits: photos A, B, C, F and I by Laura Zarraoa; photos D and G by Angela Ramón; photos E and H by Lorena Lopinto.
