# Supplementary material for: Bridging Gaps in Panthera leo Ethograms: A Systematic Review and Adaptable Model for Animal Welfare Research
Source: Animals (Basel). 2026 Oct 5;16(19):3122. doi: 10.3390/ani16193122 (PMC13663599; doi:10.3390/ani16193122)
Supplement: Supplementary file 1 [file animals-16-03122-s001.zip › Figure_S3_TailClawsLimbs_positions.pdf]

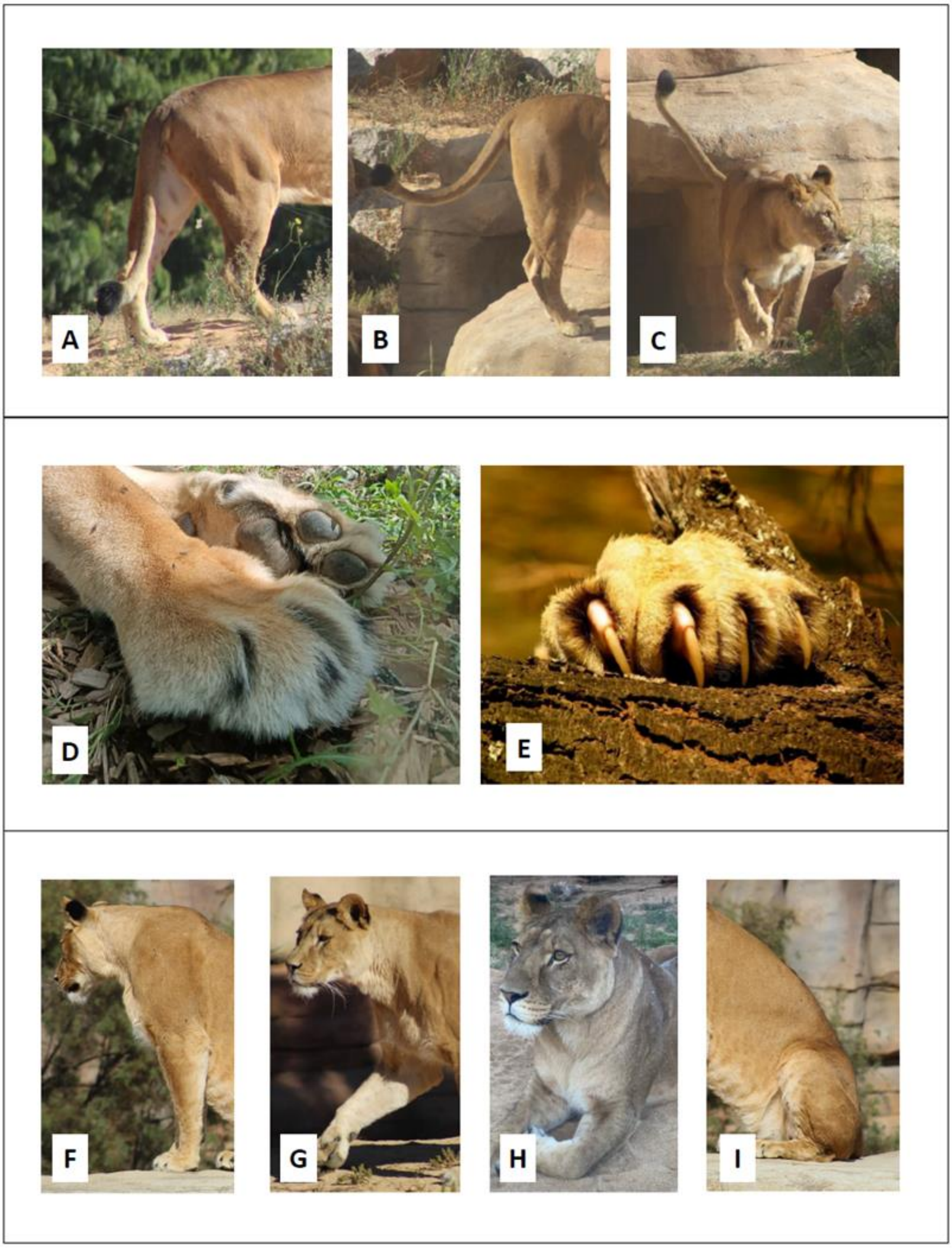

Figure S3. Positions that the tail, claws, and limbs may adopt in the *P. leo* ethogram definitions. Top: the tail may be relaxed (A), held horizontally upright (B), or held vertically upright (C). Middle: the claws may be retracted (D) or extended (E). Bottom: Extended limbs show all joints in extension, as seen in the left forelimb in the image (F). Semiflexed limbs show one joint in flexion, as observed in the elbow of the right forelimb in the image (G). Flexed limbs show two or more joints in flexion, as seen in the elbow and carpus of the right forelimb in the image (H), and in the hip, knee, and tarsus of the left hind limb in the image (I). Credits: photos A, B, C, F, G and I by Laura Zarraoa; photo D by Giovanna Donini; photo E by David Youldon; photo H by Angela Ramón.
