# Supplementary material for: Bridging Gaps in Panthera leo Ethograms: A Systematic Review and Adaptable Model for Animal Welfare Research
Source: Animals (Basel). 2026 Oct 5;16(19):3122. doi: 10.3390/ani16193122 (PMC13663599; doi:10.3390/ani16193122)
Supplement: Supplementary file 1 [file animals-16-03122-s001.zip › Lion ethogram_S4_clean.pdf]

Lion ethogram S4. Behavioural inventory for *Panthera leo*.

General guidance for ethogram use:

Behavioural categories and their corresponding patterns are organized in alphabetical order. These overarching categories complement—rather than replace—individual operational definitions by establishing shared criteria for the contained patterns. Consequently, both levels should be interpreted together.
Within the definitions, bolded and underlined terms indicate entries that are also defined elsewhere in the ethogram.
Figures S1–S3 provide the standardized anatomical and postural reference framework used throughout the operational definitions of this ethogram.
For social interactions, "actor" refers to the animal initiating the action, and "recipient" refers to the individual toward whom the action is directed.
Behaviours marked with an asterisk (*) were not observed directly, recorded on video, or identified in reviewed YouTube footage; they were adapted exclusively from the literature.
Behavioural categories were designed to be mutually exclusive to avoid overlap and double counting during data collection.
Behaviours were classified as either states (ST) or events (EV) according to the criteria described in the main manuscript. States are behaviours quantified based on duration, whereas events are brief actions quantified by frequency. Some behaviours could be classified as either states or events depending on their duration relative to the sampling resolution.
Operationalisation of event-type behaviours followed three general criteria: temporal, spatial, and postural components, as described in the Methods section of the manuscript. In this ethogram, each event is annotated with a corresponding operational code indicating the criterion used (T = temporal, S = spatial, P = postural). These codes refer to the general rules for defining behavioural occurrences and should be interpreted in conjunction with the Methods section.

Abbreviations: EE = environmental enrichment; EV = event; P = postural; S = spatial, ST = state/s; T = temporal.

Categories and behaviours:

1. Affiliative behaviours

This category includes behaviours in which the lion is close to another individual (no more than two body lengths apart) and performs actions associated with bond formation. It includes those performed by the mother towards her cubs. These interactions may occur directly or through barriers. Recipients may be another lion, a non-human animal of another species, or a human being.

Allogroom (EV or ST; T): The actor licks any part of a recipient's body, most often the head. The action may occur once or be repeated several times (Video 1).
Video 1. A lioness is observed performing two occurrences of **allogroom** toward a male lion. Simultaneously, two occurrences of **receive** are observed in the male. Between both **allogroom** events, the female performs one **self-lick** event. Credits: adapted from The Wildcat Sanctuary (2024). https://drive.google.com/file/d/1Ny_IVf59BkEK2MO0p7KZ7cDHzmDOIhOs/view?usp=sharing

Follow (EV or ST; T): The animal moves behind or beside another individual, following the same path, while maintaining a relatively constant distance. The ears are forward, the mouth closed or slightly open, and the tail remains relaxed. Various forms of locomotion may occur. No agonistic or aggressive behaviours are observed (Video 2).

Video 2. A lioness is observed performing one occurrence of **follow** with respect to another lioness. Credits: Johana Miranda Lestón.
https://drive.google.com/file/d/18uCzwIBJ66pNVaBfVzx85cORusOMyABC/view?usp=sharing

Head nudge (EV; P): The actor contacts the head of the recipient with its head using a forward movement, after which contact is broken.

Head rub (EV; P): The actor maintains contact while pressing its head along the head of the recipient (Video 3).
Video 3. Two lionesses are observed. The **standing** one (actor) performs one occurrence of **head rub**, while the **lying** one (recipient) performs one occurrence of **receive**. Credits: Walter A. D'Elia. https://drive.google.com/file/d/1WZRak-fvFjwrErneXedFRblH4syE-gCl/view?usp=sharing

Nurse* (EV or ST): The female lies in **lateral recumbency** or remains **standing** while the cubs place their mouths around her nipples and perform rhythmic sucking movements (Figure 1).

Nuzzle* (EV; T): The actor maintains contact while pressing its snout against any part of the recipient's body, except the head.

Receive (EV or ST): An individual remains in a **resting** or **standing** posture, without active intervention or **agonistic**, **fear-related**, or **aggressive behaviour**, while being the target of any affiliative behaviour emitted by another individual (Video 3).
Occurrences are counted according to the specific social behaviour received.

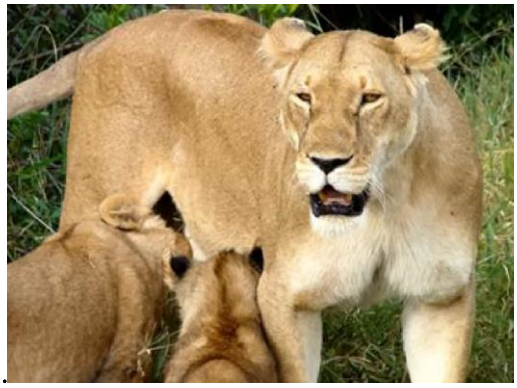

.

Fig. 1. Lioness nursing her cubs while standing.
Credits: Roel Knitel.

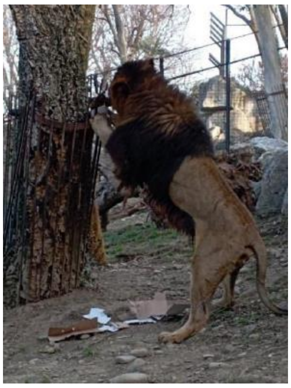

Fig. 2. The lion is performing rear up.
Credits: Giovanna Donini.

Social approach (EV; S): The actor looks at the recipient and moves toward it, reducing the distance between them. Ears are forward or backward. The mouth is closed or slightly open, with teeth barely visible. The tail is usually relaxed. No agonistic or aggressive behaviours are observed. The approach may include various forms of locomotion. (Video 4).
Video 4. A lioness is observed performing one occurrence of **social approach** toward a male lion. When the male approaches her perineal region, the female raises her tail. Credits: Walter A. D'Elia. https://drive.google.com/file/d/1AXgwrBj1ASkCiGEl0NUTk1_3MWFQg2KQ/view?usp=sharing

Social play (EV or ST; T): The actor interacts with one or more individuals using movements structurally similar to **chase**, **stalk**, **threaten** or **fight** (see **Agonistic and Aggressive behaviours**). Unlike agonistic or aggressive interactions, the mouth is half open, ears are forward or backward, the tail is relaxed and no signs of fear or injury are observed. Physical contact and vocalizations may occur (Video 5).

Video 5. A lioness and a male lion are observed performing one occurrence of **social play**. Credits: Walter A. D'Elia. https://drive.google.com/file/d/16l5xn2eF2QceTkxqnsPrUeXy0Qcpmc30/view

Social sniff (EV; T): The actor brings its snout close to any part of the recipient's body while inhaling, without necessarily making physical contact.

2. Aggressive Behaviours

This category includes behaviours in which the lion is close to another individual (no more than two body lengths apart) and performs actions associated with conflict or competition involving physical contact. Recipients may include another lion, another non-human animal species, or a human.

Fight (EV or ST; T): The animal directs swipes and bites at another individual, involving physical contact. Physical interactions may also include grappling or wrestling movements between opponents. It may be **standing**, **rearing up** on the hindlimbs with the forelimbs placed on an opponent, or positioned in any **recumbent** posture. The ears are held back or flattened, the tail is erect, and the mouth is open, displaying the teeth. Vocalisations are produced. The dorsal fur is often piloerect. Two or more animals may be involved (Video 6).

Tackle (EV): The actor collides with another individual following a **run** or **jump** and places its forelimbs on or around the recipient. This behaviour often precedes **fight** (Video 6).
Video 6. A lion is observed performing **tackle** towards another lion, followed by **fight**. Credits: adapted from Knitel (2016).
https://drive.google.com/file/d/1ZVqQ06ajXxoulKZLW4Iu1R46MdWe4pvW/view?usp=sharing

3. Agonistic Behaviours

This category includes behaviours in which the lion is close to another individual (no more than two body lengths apart) and displays actions associated with conflict or competition, without physical contact. These behaviours may occur directly or through barriers. Recipients may include another lion, a non-human animal of another species, or a human being.

Displace* (EV; S): The actor approaches or changes its position/orientation in such a way that it provokes an avoidance response from the receiver (**retreat** or **social flee**).

Flatten (EV; P): Upon the presence of another individual, the animal bends all four limbs, bringing the belly close to the ground. It may remain **crouched** or lie in **lateral recumbency** (Videos 7 and 8). It can be accompanied by **threaten** or **submit**, without a specific order.
Video 7. A lioness is observed simultaneously performing **flatten** and **threaten** behaviours in the presence of a lion. Credits: Walter A. D'Elia.
https://drive.google.com/file/d/1ajjdUs9SO3ZcIcikMcfK4yQuPFiDzXrX/view?usp=sharing

Retreat* (EV): The animal **walks** away from another individual after a previous agonistic (e.g., **threaten** or **displace**) or aggressive (**fight**, **tackle**) interaction. If no such interaction is observed, the behaviour is classified as **walking** (see **Locomotory behaviours**).

Social flee* (EV): The animal **runs** or **trots** away from another individual after a previous agonistic (e.g., **threaten** or **displace**) or aggressive (**fight**, **tackle**) interaction. If no such interaction is observed, the behaviour is classified as **running** or **trotting** (see **Locomotory behaviours**).

Social pursue* (EV; T): The animal **runs** after another individual. If the target is prey, the behaviour is classified as **chase prey** (see **Feeding behaviours**).

Submit (EV; P): The animal rolls onto its back and remains still, exposing its belly, with all four legs raised. Teeth are usually visible and claws extended. It may be accompanied by vocalizations and ears held back or flattened.

Threaten (EV; T): The actor opens its mouth with the lips retracted, exposing the teeth (including the incisors and canines), while the skin of the upper part of the snout is wrinkled. It may also strike the ground with its fore or hindlimbs, swats, and produces various vocalizations, without making physical contact with the recipient. Ears are usually held back or flattened (Videos 7 and 8).
Video 8. A lioness is observed performing **threaten** followed by **flatten** in the presence of a lion. Credits: Walter A. D'Elia.
https://drive.google.com/file/d/1DKNg-oYAsE0OfK7BrmtSWnpP42raseIp/view?usp=sharing

4. Environment-directed behaviours

Behaviours in which the individual investigates the surroundings or a specific stimulus. This category includes behaviours directed toward environmental enrichment. It does not include marking behaviours nor behaviours directed toward other individuals (see **Affiliative**, **Aggressive**, or **Agonistic behaviours**).

Chew object (EV or ST; T): The animal crushes or breaks a non-edible object in its mouth through jaw movements.

Dig (EV; T)*: The animal performs alternating movements with its forelimbs, breaking up or displacing the substrate.

Explore (EV or ST; T): The animal sniffs the ground, the air, or objects while **walking**, or by making pauses of up to 3 s during locomotion (Video 9).
Video 9. A lion performing an occurrence of **explore**. Credits: Walter Adrián D'Elia. https://drive.google.com/file/d/10TBEC8Qu6ZisSd9UToW8JyC3mGW3pAtz/view?usp=sharing

Flehmen (EV; T): The animal adopts a characteristic facial expression in which the mouth is open, the upper lip is lifted, and the tongue may protrude. It usually follows **sniffing** an object or an odour or body secretion from another individual (Video 10).
Video 10. A lion performing **sniff** followed by **flehmen**. A lioness is also seen performing **sniff**. Credits: Giovanna Donini y Ramiro Rodriguez.
https://drive.google.com/file/d/1upOnqy1Eq65eQkXaYsDDJeNwAqHk16X-/view?usp=sharing

Lick* (EV; T): The animal passes its tongue over an inanimate surface or object (e.g., ground, enrichment stimulus), either once or repeatedly. If the action is directed toward its own body, it is recorded as **self-groom** (see **Maintenance behaviours**); if directed toward another individual, it is recorded as **allogroom** (see **Affiliative behaviours**).

Manipulate (EV or ST; T): The animal uses part of its body—usually the forepaws or the mouth—to touch, hold, move, or pick up an object (Videos 11 and 12).
Video 11. A lion performing **manipulate** directed at an EE object (paper bag). Credits: Walter Adrián D´Elia.
https://drive.google.com/file/d/1W6nOcoR50UliC1Thu5GnRFeOXFANmDlv/view?usp=sharing
Video 12. A lioness performing **manipulat**e directed at an EE object (cardboard box). Credits: Giovanna Donini y Ramiro Rodriguez.
https://drive.google.com/file/d/1qEwcuMngW8D6WVJSESnryNbQr1J7odpB/view?usp=sharing

Stalk object (EV or ST; T): The animal moves while keeping its head lowered in relation to the back. Its gaze remains fixed on an object. The ears are oriented towards the target or may be held back. The belly may move closer to the ground.

Sniff (EV; T): The animal remains still and brings its snout close to an object or surface while inhaling. It may also sniff the air, with its head raised and without directing the snout towards any specific surface (Videos 10 y 13). If performed while **walking**, it is recorded as **explore**. If directed toward another individual, it is recorded as **social sniff** (see **Affiliative behaviours**).
Video 13. A lion is observed performing the **sniff** behaviour. Credits: Walter Adrián D ´Elia. https://drive.google.com/file/d/1JSeQXfbPFtVCnPam3kRJ3t-9AQIMa_Pz/view?usp=sharing

Rear up (EV or ST; P): The animal rises up onto its hind legs, with the body in a vertical or oblique position, while the forelimbs remain free or resting against an object (Figure 2).

5. Fear-related behaviours

Behaviours characterized by avoidance responses, immobility, and heightened vigilance, commonly associated with situations involving actual or perceived threats or environmental disturbances.

Flee* (EV): The animal **runs** away from a potentially threatening stimulus. If the stimulus is another individual, this behaviour should be classified as **social flee** (see **Agonistic behaviours).**

Freeze* (EV or ST): The animal interrupts its current behaviour and remains motionless, with an upright body posture, ears forward, and gaze fixed. The tail may be tucked under the abdomen, and the animal may tremble.

6. Feeding behaviours

Any behaviour associated with the acquisition and ingestion of food or water, including feeding enrichment.

Attack prey (EV): The animal **jumps** onto a prey item (Video 14).
Video 14. A lioness is observed performing a hunting sequence. The video illustrates how several behaviours included as independent categories in the ethogram may occur sequentially during predation. In this example, the sequence consists of **stalk prey**, **chase prey**, **attack prey**, **capture**, and **kill**. Credits: Adapted from National Geographic España (2022).
https://drive.google.com/file/d/1Wdx_z8RbuS3fMafvlwu6-vUXpTuGVAsD/view?usp=sharing

Capture (EV): The animal secures the prey item using its claws and/or mouth (Video 14).

Chase prey (EV): The animal **runs** after a prey item (Video 14).

Chew food (EV or ST; T): The animal performs mandibular movements while holding an edible item in its mouth (Video 15).
Video 15. A lion is seen performing an occurrence of **chew food** with a piece of meat containing bone.
Credits: Walter A. D'Elia.
https://drive.google.com/file/d/12DaTUtNs6zB88zCLeapH-HJyCMwWUrCH/view?usp=sharing

Drink (EV; T): The animal brings its head close to a liquid and ingests it using repeated tongue movements (Video 16).
Video 16. A lioness is observed performing an occurrence of **drink**. Credits: Giovanna Donini and Ramiro Rodriguez.
https://drive.google.com/file/d/1wivdnAbGbEOBJv7kLMkhnVWWiv3QT6FT/view?usp=sharing

Kill (EV): The animal bites the prey's neck or covers its snout with its own mouth (Video 14).

Stalk prey (EV or ST; T): Same as **stalk object** but directed toward prey (Video 14).

Suckle* (EV or ST; T): The cub places its mouth around a female's nipple and remains attached while performing repeated rhythmic movements of the muzzle and jaw (Figure 1).

Transport food (EV; T): The animal moves food (typically a prey item or part of it) from one place to another. This may involve dragging it along the ground while holding it in the mouth, or carrying it entirely off the ground (Video 17).
Video 17. A lion is observed performing an occurrence of **transport food** with a piece of meat containing bone. Credits: Walter A. D'Elia.
https://drive.google.com/file/d/1WlJRONoZ-czfCxReQ6xRoZrTS5GixzNs/view?usp=sharing

7. Locomotor behaviours

Behaviours that enable movement from one place to another. This category provides the operational definitions for the basic locomotor patterns used throughout the ethogram. Although these patterns form the basis of several more complex behaviours, they should be recorded within this category only when they occur as standalone actions. When a locomotor pattern occurs simultaneously with another behaviour (e.g., **explore**) or within a specific behavioural context (e.g., **follow**, **chase prey**, or **reproductive evasion**), it should be recorded according to that behaviour's operational definition to ensure mutual exclusivity and prevent double counting.

Ascend (EV): The animal moves onto an elevated surface. It first places its forelimbs on the upper level and then pushes the body upward extending the hindlimbs until all four paws are supported on the elevated surface (Video 18).
Video 18. A lioness is seen performing an occurrence of **ascend**. Credits: Walter A. D´Elia. https://drive.google.com/file/d/1GDvDkOQL3Q_bouw7cQbj2u0Y16YUjxF_/view?usp=sharing

Climb (EV; T): The animal moves upward or along a vertical or inclined surface, gripping with extended claws and propelling itself through the coordinated flexion and extension of the limbs.

Descend (EV): The animal moves down from an elevated surface by flexing and extending its limbs. It first places the forelimbs on the lower level and then lowers the rest of the body until all four paws are on the ground (Video 19).
Video 19. A lioness is seen performing an occurrence of **descend** from a rock. Credits: Walter A. D´Elia. https://drive.google.com/file/d/1Xf4P52S4ezSFq3Q3K6_CS4TOTvKtPVVr/view?usp=sharing

Jump (EV): The animal propels itself by simultaneously extending the hindlimbs and lifting all four paws off the surface, moving from one point to another—either vertically or horizontally—until the paws contact the surface again (Video 20). If the action is directed toward a prey item, it is recorded as **attack prey** (see **Feeding behaviours**); if it is directed toward landing on another individual, it is recorded as **tackle** (see **Aggressive behaviours**).
Video 20: A lioness is seen performing an occurrence of **jump**. Credits: Giovanna Donini y Ramiro Rodriguez. https://drive.google.com/file/d/1gLW2kStvnoEEUCHHEi_OUGjiUnsEWGT-/view?usp=sharing

Run (EV; T): The animal moves forward by alternating the movement of its forelimbs and hindlimbs. The hindlimbs make ground contact one at a time and propel the body forward while the forelimbs extend forward. Then, the forelimbs make ground contact one at a time while the hindlimbs rise. Finally, the hindlimbs cross beneath the body with the forelimbs, followed by an aerial phase (no paws touching the ground) (Video 21).
Video 21. A lioness is seen performing an occurrence of **run**. Credits: Walter A. D´Elia. https://drive.google.com/file/d/1H91oqtLk1tA9YIgtYbEjYShZMPY28HYl/view?usp=sharing

Swim* (EV; T): The animal propels itself through water using rhythmic and coordinated movements of all four limbs. The head is usually kept raised above the water surface.

Trot (EV; T): The animal moves forward using a diagonal, alternating gait pattern (right hindlimb with left forelimb, and vice versa) (Video 22).
Video 22: A lioness is seen performing the **trot** behaviour. Credits: Walter A. D´Elia. https://drive.google.com/file/d/1QmcayfzLXQSTls3dAX_5F9tiY1qjZ-35/view?usp=sharing

Walk (EV or ST; T): The animal moves forward by lifting and advancing the limbs on one side of the body (forelimb and hindlimb on the right side, or on the left side) and then alternating with the limbs on the opposite side (Video 23). At slow speeds, one limb may be advanced at a time while up to three limbs remain in contact with the ground.
Video 23. A lion is seen performing the **walk** behaviour. Credits: Walter A. D´Elia. https://drive.google.com/file/d/1aLtFVzed6a89BoldnQqUu7C-xmTIjfeO/view?usp=sharing

8. Maintenance behaviours

Behaviours associated with physiological needs and self-care, excluding "Feeding behaviours" and certain "Marking behaviours".

Self-scratch (EV; T): The animal rubs one of its paws against some part of its own body, generally repeatedly (Video 24).
Video 24. A lioness is observed performing a **self-scratch** occurrence, followed by **head shake**.
Créditos: Giovanna Donini y Ramiro Rodriguez.
https://drive.google.com/file/d/1CV6pShI13KGSoMHQVQz5QCja1Ixmzo6A/view?usp=sharing

Self-groom (EV or ST; T): The animal licks or bites any part of its own body (Video 25).
Video 25: A lioness is observed performing an occurrence of **self-groom**. Credits: Walter A. D'Elia. https://drive.google.com/file/d/1W3LBdPZvBeZcCw8yjNkHc_9kF6VIkfyy/view?usp=sharing

Stretch (EV): The animal extends its forelimbs while arching its back downward, lowering the abdomen toward the ground and raising the head and the hindquarters (Video 27). This may be accompanied by **head shake** (Videos 24 and 26) and **yawn** (Videos 26 and 27).
Video 26. A lion is observed performing a **stretch** occurrence. It is then observed performing **yawn** and finally **head shake**. Credits: Walter A. D'Elia.
https://drive.google.com/file/d/1ZMS0liRD_Ehi8UwuBiLHi6RyR-PklTlw/view?usp=sharing

Urinate (EV; P): The animal releases urine onto the ground while keeping the hindlimbs flexed, the hindquarters lowered, and the tail raised horizontally. This may be preceded or followed by **scraping with the hind paws**.

Yawn (EV): The animal opens its mouth, exposing the teeth, and extends the tongue while inhaling. It often also extends the neck and raises the head. It then closes the mouth while exhaling (Videos 26 and 27).
Video 27. Two lionesses are observed resting in **sternal recumbency**. The one in the back performs a "Yawn" occurrence. Credits: Walter A. D'Elia.
https://drive.google.com/file/d/1ElH398K_MajGVgruV9SzyyXbGjwr2kma/view?usp=sharing

9. Marking behaviours

Behaviours related to communication between individuals, through which olfactory or visual signals are deposited on a surface, generally for territorial, reproductive, or social purposes.

Defecate (EV; P): The animal expels faeces onto the ground or into water while keeping the hindlimbs flexed, the hindquarters lowered, and the tail raised horizontally. This may be preceded or followed by scraping with the hind paws.

Rub object (EV; P): The animal presses and slides a part of its body—generally the head, neck, or flanks—against an object or surface (Video 28).
Video 28. A lion is observed performing a **rub object** occurrence against a log. Credits: Giovanna Donini y Ramiro Rodriguez.
https://drive.google.com/file/d/19VRJN1m0TlGeM9JoMu0-GuSfz07SEAvE/view?usp=sharing

Scrape with hind paws (EV; T): The animal drags the hindlimbs backward over the ground, alternately and repeatedly. This action may displace some vegetation, soil, or other substrate material. It often accompanies other behaviours such as **defecate**, **urinate**, **spray**, or **rub objects** (Video 29).
Video 29. A lion is observed performing a **scrape with hind paws** occurrence. Credits: Walter A. D'Elia.
https://drive.google.com/file/d/1FAdc1PLXW1f4WJEtx2L0I1fUoI1DsjbY/view?usp=sharing

Scratch (EV; T): The animal extends the claws of the forelimbs and drags them over an object or surface, potentially leaving visible marks (Video 30).
Video 30. A lioness is observed performing a **scratch** occurrence on a log. Credits: Giovanna Donini y Ramiro Rodriguez.
https://drive.google.com/file/d/1L7H012tFXSj1AHkT4IHM4trhV537Cxhl/view?usp=sharing

Spray (EV): The animal **stands** with the tail raised vertically and directs a jet of urine backward onto a vertical surface or object. The tail may tremble during the release of urine (Video 31).
Video 31. A lion is observed performing a **spray** occurrence against a tree. Credits: Walter A. D'Elia. https://drive.google.com/file/d/1iZu7SsI29udUW3-BXL6tlPjmuQrKElyU/view?usp=sharing

10. Not observable

This is not a category or a behaviour, but an annotation that must be recorded when the focal animal is not visible, or is only partially visible, making it impossible to accurately determine which behaviour it is performing.

11. Other behaviours

Behaviours that do not fit into any of the defined categories. This category includes actions that cannot be reliably classified elsewhere due to their ambiguity, rarity, or context-dependent nature.

Air bite (EV; T): The animal raises its head and opens and closes its mouth. This behaviour is usually directed at flying insects (Video 32).
Video 32: A lion is observed performing an occurrence of **air bite**. Credits: Giovanna Donini y Ramiro Rodriguez.
https://drive.google.com/file/d/1jJw0gaeGaMpElQl6r6X5MD8x6RuVXlYA/view?usp=sharing

Crouch (EV or ST): The animal remains with its belly close to the ground, limbs flexed, and the elbows and/or knees touching the ground. The head may be aligned with the body or slightly raised, and the tail may be still or moving slowly. The eyes remain open and fixed on a stimulus.

Head shake (EV): The animal rotates its head laterally, moving it from side to side in a horizontal plane. This typically consists of 1 to 10 movements lasting no more than 3 seconds. The ears often strike the top of the head during the movement (Videos 24, 26 and 33).
Video 33. A lioness is observed performing the **head shake** behaviour. Credits: Giovanna Donini y Ramiro Rodriguez.
https://drive.google.com/file/d/1P2mJbNORLzy50F-tAbz1f2uqKB-6-W1-/view?usp=sharing

Lip lick (EV; T): The animal passes its tongue over its lips or snout. It may occur in association with feeding, exploratory or reproductive behaviours, or appear in isolation (Video 34).
Video 34: A lioness is observed performing an occurrence of **lip lick**. Credits: Giovanna Donini y Ramiro Rodriguez.
https://drive.google.com/file/d/10O9lMKxoexh4cMMILOwbesQ2qeWAKhQx/view?usp=sharing

Mount* (EV or ST): The animal positions itself over another individual from behind, by straddling it with the fore and hindlimbs. This behaviour may occur between opposite-sex or same-sex individuals and may or may not lead to **copulation**.

Stalk individual (EV or ST): Same as **stalk object** but directed toward another individual located more than two body lengths away. If the target is prey, it should be classified as **stalk prey** (see **Feeding behaviours**).

Stand (ST): The animal remains upright, with the longitudinal axis of the body parallel to the ground, all four paws on the floor, and all limbs extended (Figure 3).

Vocalize (EV; T): The animal emits audible sounds that may vary in pitch and duration.

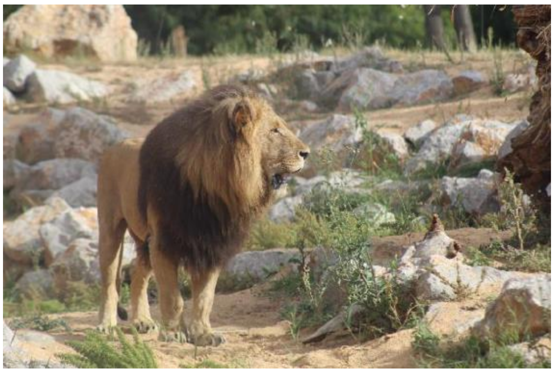

Fig. 3. Standing lion. Credits: Laura Zarraoa.

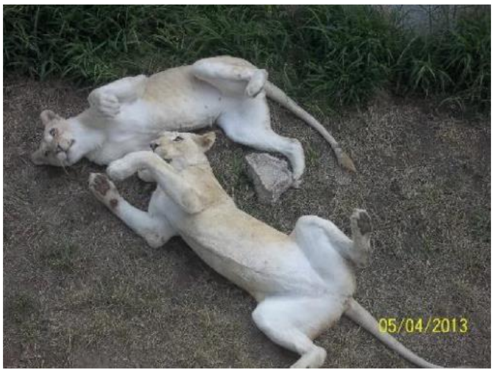

Fig. 4. Lionesses resting in dorsal recumbency. Credits: Carola Milozzi

12. Potentially stereotypic behaviours

Repetitive behavioural patterns with low or no variability. The behaviour must be performed three or more times consecutively for it to be considered stereotypic.

Head move* (EV or ST; P): The animal **stands** or **sits** and moves its head in a circular pattern, up and down, or side to side.

Forepaw lift* (EV or ST; T): The animal **stands** and alternately lifts its forelimbs, raising one while the other remains on the ground.

Pace (EV or ST; P): The animal **walks** following a fixed pattern, moving back and forth along the same route. Head movements may be included (Video 35).
Video 35. A lioness is seen performing four repetitions of **pace**. Credits: Walter A. D ´Elia. https://drive.google.com/file/d/1Lt0nPFHhnEWN0k51YoTWQCCcYmsPzWem/view?usp=sharing

Self-mutilate* (EV or ST; T): The animal performs behaviours directed toward its own body—such as **licking**, **biting**, or pulling out hair—that result in injuries or damage to the skin and coat.

Stereotypic snout rub* (EV or ST; P): The animal moves its head from side to side while rubbing its snout against a vertical surface.

Stereotypic rub* (EV or ST; P): The animal presses and slides part of its body against a vertical surface. The rubbing often leaves visible marks on the surface and may cause hair loss on the contacted body areas.

Sway* (EV or ST; T): The animal **stands** and rocks its body from side to side.

13. Reproductive behaviours

Sexual behaviours associated with mating and reproduction.

Copulation* (EV): The actor remains **mounted** and performs pelvic thrusting movements. It may also place its mouth around the recipient's nape. The termination of the interaction is typically followed by **post-copulatory rolling** or **post-copulatory anogenital grooming**.

Post-copulatory anogenital grooming* (EV or ST): The animal licks its anogenital area after the copulatory act.

Post-copulatory rolling* (EV): The female **threatens** the male and then rolls and moves away. It is usually observed 5 to 10 seconds after the female emits the "copulatory cry" (Stanton et al., 2015).

Reproductive evasion* (EV or ST): The female escapes from the male by **running** or **trotting**. She then stops to roll or look around before continuing to flee when the male approaches. Both members of the pair may also **walk** around or circle each other. It is usually preceded by other reproductive behaviours and precedes the male's **mount**.

14. Resting behaviours

ST in which the individual remains immobile or with minimal movement, without displacement. If during rest the individual simultaneously performs an activity assigned to another category (e.g., "milk"), it will be recorded according to the corresponding category, and not as rest.

Alert resting (ST): The animal is in any of the **resting** or **standing** behaviours. The head is upright, and the mouth is closed or slightly open. The ears are directed forward, either still or moving. The eyes are open, alternating between focusing on specific elements or moving from one point to another in the environment. If the observed element moves, the animal may follow it with head movements (Video 36).
Video 36. Two lionesses observed in **alert resting**. Credits: Walter A. D'Elia. https://drive.google.com/file/d/1wZEMe2a566TFWbK580_27F5Y5vCXzQZJ/view?usp=sharing

Dorsal recumbency (ST): The animal lies on its back with the abdomen exposed. The head rests on the ground, and the limbs may be flexed, semi-flexed, or extended. The eyes are usually half-open or open (Figure 4).

Lateral recumbency (ST): The animal lies on its side, with one flank in direct contact with the ground. The head may be upright or resting, and the limbs may be flexed, semi-flexed, or extended. The eyes are usually half-open or closed (Figure 5).

Perching (ST): The animal lies with its abdomen resting on a branch or log while at least one limb is extended downward, hanging in the air. The head may be upright or resting (Figure 6).

Sitting (ST): The animal is positioned with all four paws on the ground, the hindlimbs flexed, the anogenital region resting on the ground, and the forelimbs extended (Figure 7).

Social resting (ST): The animal is in any resting behaviour, with some part of its body in contact with one or more individuals, but without active social interaction behaviours (Figure 8).

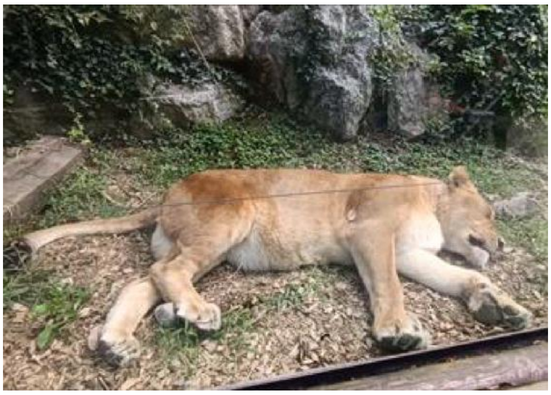

Fig. 5. Lioness resting in lateral recumbency. Credits: Giovanna Donini.

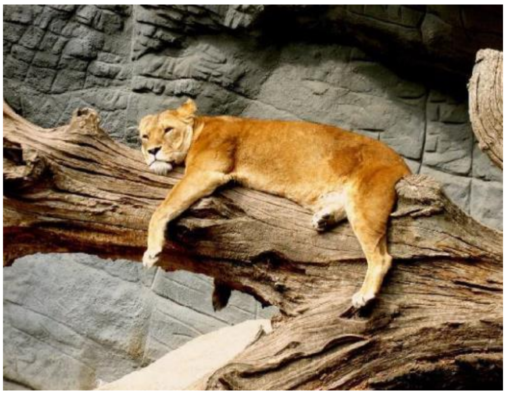

Fig. 6. Lioness perching. Credits: Mona El Falaky (Pixabay).

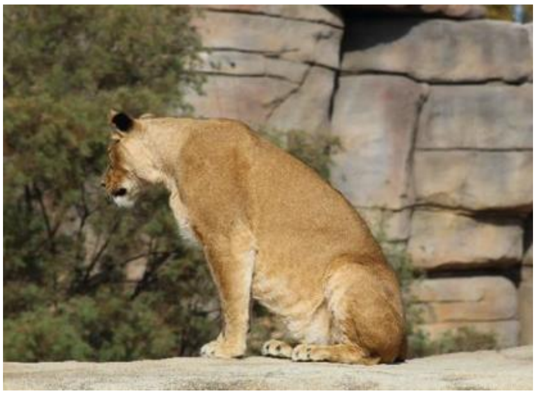

Figure 7. Lioness sitting. Credits: Laura Zarraora.

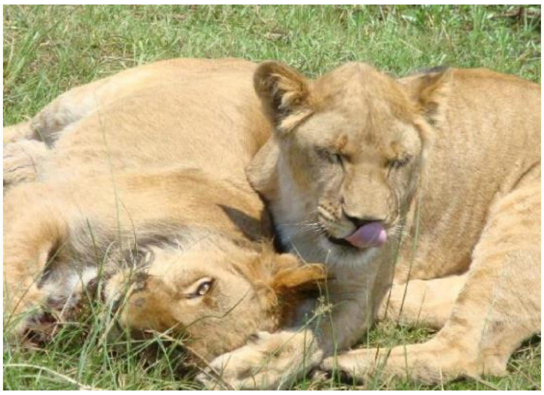

Figure 8. Juvenile male lion and lioness in social rest. Credits: Rocío Lopinto.

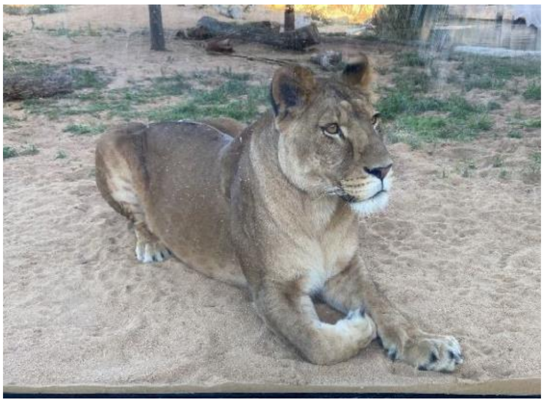

Fig. 9. Lioness resting in ventral recumbency, with the left forelimb extended and the right forelimb and both hindlimbs flexed. Credits: Angela Ramón.

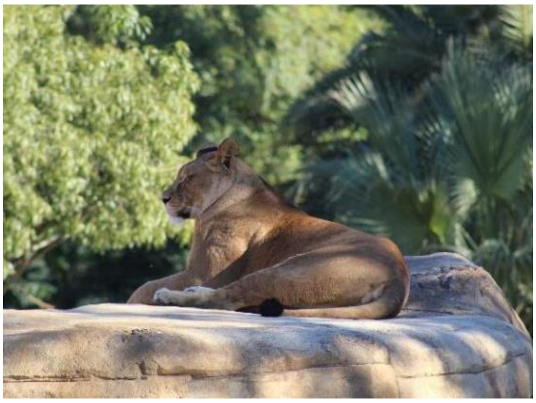

Fig. 10. Lioness resting in ventral recumbency, with the hindlimbs extended to one side. Credits: Laura Zarraoa.

Ventral recumbency (ST): The animal lies on its abdomen, on the ground or a flat surface, with all limbs either supported by the substrate or flexed beneath/beside the body. The forelimbs may be flexed, semi-flexed, or extended forward, while the hindlimbs may be flexed, semi-flexed, or extended to one or both sides. The head may be upright or resting on the forelimbs (Figures 9 y 10). If at least one limb is extended downward, hanging in the air from a branch or log, the behaviour is classified as **perching**.
